# Short tryptophan- and arginine-rich peptide shows efficacy against clinical methicillin-resistant *Staphylococcus aureus* strains isolated from skin and soft tissue infections

**DOI:** 10.1038/s41598-019-53926-4

**Published:** 2019-11-20

**Authors:** Mihaela Bacalum, Elena-Carmina Dragulescu, George Necula, Irina Codita, Mihai Radu

**Affiliations:** 1 Department of Life and Environmental Physics, “Horia Hulubei” National Institute of Physics and Nuclear Engineering, Măgurele, Romania; 2 Nosocomial and Antimicrobial Resistant Infections Laboratory, “Cantacuzino” National Medico-Military Institute for Research and Development, Bucharest, Romania; 3 Department of Computational Physics and Information Technologies, “Horia Hulubei” National Institute of Physics and Nuclear Engineering, Măgurele, Romania; 4 “Carol Davila” University of Medicine and Pharmacy, Bucharest, Romania

**Keywords:** Antimicrobial resistance, Clinical microbiology

## Abstract

In recent years methicillin-resistant *Staphylococcus aureus* has posed a challenge in treating skin and soft tissue infections. Finding new antimicrobial agents has therefore become imperative. We evaluated the *in vitro* antimicrobial activity of a synthetic peptide, P6, against multidrug resistant clinical strains of *Staphylococcus aureus* isolated from skin and soft tissue infections. The P6 antimicrobial effect was evaluated *in vitro* by determining MIC/MBC, the ratio of live/dead cells and the effects induced at membrane level. The therapeutic efficiency was determined against human skin cells. P6 inhibited growth for all strains between 8 and 16 mg/L and killed all bacterial strains at 16 mg/L. The therapeutic potential was found to be 30 and 15 in the presence of BSA. We showed that P6 localizes at membrane level, where it acts slowly, by depolarizing it and affecting its integrity. P6 can be considered a good candidate for use as an antimicrobial agent in topical applications.

## Introduction

*Staphylococcus aureus* (*S*. *aureus*) is a ubiquitous bacterium commonly colonizing the skin and mucosal surfaces of humans, several animal and avian species. However, it may become a dreadful cause of skin and soft tissue or invasive infections, like bacteremia, infective endocarditis, osteoarticular infections, prosthetic device infections etc., especially in immunocompromised patients^1^. Antimicrobial resistance, which developed progressively under the pressure of antibiotic use and other environmental factors is nowadays considered by the World Health Organization “a growing public health threat of broad concern to countries and multiple sectors”^2^. Particularly, methicillin-resistant *S*. *aureus* (MRSA) poses a challenge in treating skin and soft tissue infections (SSTIs)^3,4^. The most epidemiologically important sites of colonization are the nasal cavity, throat, perineum and skin, with a rate of nasal carriage of *S*. *aureus* varying between 20 and 30% of the population^5^. Another problem also arises from its ability to adhere to the extracellular matrix and plasma proteins deposited on biomaterials^6^ and to form biofilms^7^ which enables bacteria to escape environmental stressors. An aggravating factor is the decrease in the pace of evaluation and the marketing of new antibiotics active against bacteria resistant to existing antibiotics, which led to evoking the possibility of returning to the pre-antibiotic era. These issues have catalyzed scientific knowledge-oriented concerns to substantiate the identification of new therapeutic modalities and/or to limit the proliferation of resistant microorganisms^8^.

Considering these aspects, it was stated that reducing the bioburden in the hospital environment is critical for reducing the risk of infection for people in contact with that environment^9^. A negative consequence of current antibacterial agents used as antiseptics or disinfectants is their contribution to rising antimicrobial resistance and equally harming the environment by disrupting the ecological balance of soils and aquatic life^10^.

Several research groups have taken action in response in order to develop antimicrobial alternatives to antibiotics, antiseptics and disinfectants, as novel compounds with antimicrobial activity and antimicrobial substances, including nanostructured surfaces targeting the disruption of bacteria through physical rather than biochemical interactions^11^.

Antimicrobial peptides (AMP) are recognized as a promising class of substances having high potential for biotechnological applications. Their particular physical features (negative charge, hydrophobicity, etc) allow for specific mechanisms of action, mainly damaging the bacterial membrane^12^. In the last decade many natural and synthetic peptides have been studied with respect to their bactericide effects on *S*. *aureus* strains (for a review see^13^). However, in a very recent report focused on the analysis of the clinical trials devoted to antimicrobial approaches against *S*. *aureus*, the antimicrobial peptides are included with only one, unsuccessful, clinical trial^14^. The main drawback of the tested AMP structures is the low therapeutic index leading to a high level of side effects in clinical trials. New AMP structures that exploit better the main advantages of AMP could be of real help in finding a feasible solution against *S*. *aureus*. The short tryptophan rich cationic peptides, like the highly studied indolicidin (a 13 amino-acid peptide isolated from bovine neutrophils, positively charged, 5 tryptophan residues), have been proposed as possible efficient bactericides^15^. Aside tryptophan residues, a high antimicrobial efficiency against MRSA was also proved for small peptides containing arginine and lysine residues^16–19^. In a recent report we proposed a synthetic short tryptophan and arginine rich peptide as a candidate for antimicrobial approaches^20^. The peptide (namely P6, structure HRWWRWWRR) proved good efficiency against Gram negative and positive bacterial strains (including *S*. *aureus*).

In this study we investigated the antimicrobial effect of P6 on several multidrug resistant clinical strains of *Staphylococcus aureus* isolated from skin and soft tissue infections. In accordance with our results, a possible action mechanism is outlined, also future clinical applications were considered.

## Results

### Determination of MIC and MBC values

The antimicrobial activity of P6 against 10 clinical *S*. *aureus* strains of which 9 were MRSA (A1-A9) and one Methicillin-Susceptible *Staphylococcus aureus* (MSSA) (A10) is presented in Table 1. The activity against an *S aureus* reference strain was also included, according to data reported previously^20^. For all strains the bacteriostatic effect was observed at concentrations between 8 and 16 mg/L, similarly to the results previously obtained on the reference strain^20^. No direct correlation was observed between the MIC values and the antibiotic resistance patterns. MBC for all strains was the same: 16 mg/L.

**Table 1:** Bacteriostatic (MIC) and bactericidal (MBC) concentration of P6 against the clinical S. aureus strains.

Strain code	Antibiotics resistance pattern^4^	MIC (mg/L)	MBC (mg/L)
A1	P, FOX, K, TE	8	16
A2	P, FOX, E, DA (MLSBi), K, TE, QD	16	16
A3	P, FOX, E, DA (MLSBi), K, QD	12	16
A4	P, FOX, E	12	16
A5	P, FOX, E, DA, K, CIP, TE, C	8	16
A6	P, FOX	8	16
A7	P, FOX, E, K, CIP	16	16
A8	P, FOX, K, TE, FD	12	16
A9	P, FOX, E, DA (MLSBi), K, TE, C, QD, FD	16	16
A10	P	12	16
Reference strain (ATCC 6538)	NA	12*	12*

P - benzylpenicillin, FOX - cefoxitin, E - erythromycin, DA – clindamycin, K - kanamycin, CIP - ciprofloxacin, TE – tetracycline, C - chloramphenicol, QD - quinupristin/dalfopristin, FD - fusidic acid, MLSBi phenotype - inducible resistance to macrolides, lincosamides and streptogramin B phenotype. *The MIC and MBC for the reference strain have been measured in a previous study^20^.

### BSA, serum and salts inhibition effect on peptide activity

In order to explore the potential of P6 for clinical application the MIC and MBC values were measured for bacteria grown in the presence of 4% BSA (see Supplementary Data), 25% or 50% FBS and 100 or 200 mM NaCl. We found that the MIC value increases 2 times after BSA addition into media. BSA and P6 interaction was also studied by fluorescence spectroscopy and molecular simulation, the data suggesting a rapid binding of P6 on BSA functional sites (see Supplementary Data). FBS addition inhibited even further the P6 activity, with 25% of FBS, the MIC value was found at 128 mg/L, while 50% of FBS inhibited P6 activity even at 128 mg/L. For salt addition, independent of the concentration added, P6 inhibited bacterial growth at 32 mg/L.

### Cytotoxicity assay -Therapeutic Index (TI)

**Figure 1:**
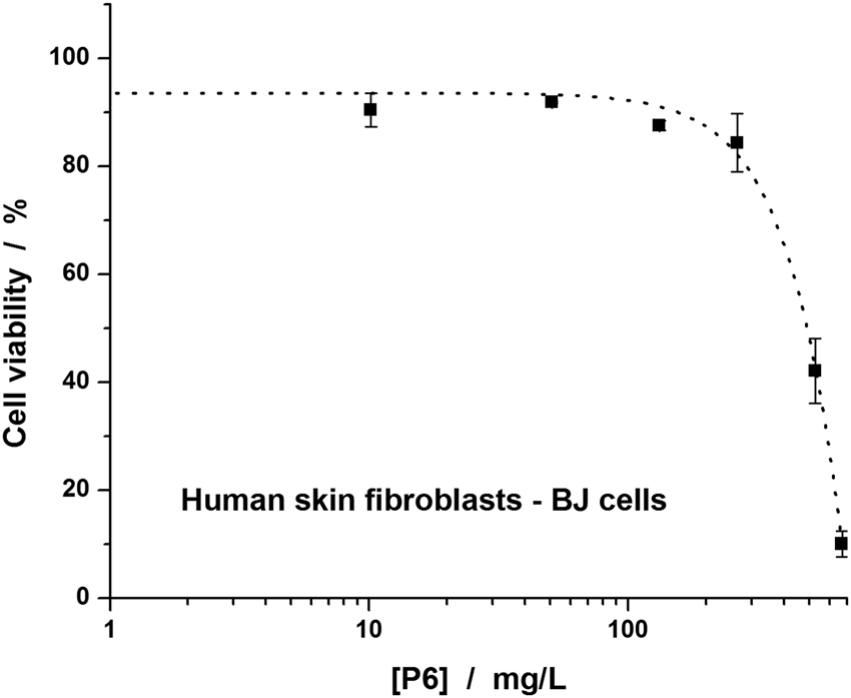
*In vitro* toxicity of P6 on human skin fibroblasts.

P6 cytotoxicity was assessed on human skin fibroblast BJ cell line for a range of concentrations up to ~700 mg/L (Fig. 1). The peptide affects the viability of the cells in a dose dependent manner, decreasing the viability down to 10% for the highest concentration tested. IC_50_ value was determined at 490 mg/L. To characterize the P6 therapeutic potential against MRSA skin and soft tissue infections the therapeutic index (TI) was calculated as the ratio of IC_50_ over MIC^21^ a value of ~30 being inferred for standard growing conditions. However, addition of salts or 4% BSA reduced twice the TI, while the serum (25%) strongly decreased the TI down to ~3.

### Live/dead assay

**Figure 2:**
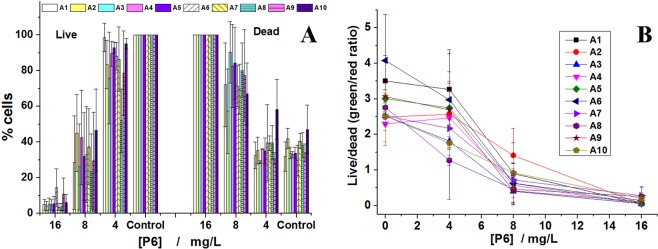
Percentages (**A**) and ratios (**B**) of live and dead bacteria treated with different concentrations of P6.

We also checked the percentages of live and dead bacteria treated with P6 at concentrations of 16, 8 and 4 mg/L representing MBC, MBC/2 and MBC/4 for all 10 clinical bacterial strains (Fig. 2A). When treated with 16 mg/L of P6 all bacteria were dead. For a concentration of 8 mg/L, the percentage of dead cells is lower and the fluctuations from one strain to another correlate qualitatively with the MIC distribution presented in Table 1, while for the bacteria treated with 4 mg/L the percentage of dead cells is similar to that of untreated bacteria. In Fig. 2B we presented the ratios of live and dead bacteria for different concentrations of P6. The most efficient was the treatment with 16 mg/L, while for some of the bacterial strains, 8 mg/L also induced similar results as for 16 mg/L. Even so, at 4 mg/L the dead/live ratio for all bacterial strains was similar to the one obtained in the case of untreated bacteria.

### Transmembrane potential

**Figure 3:**
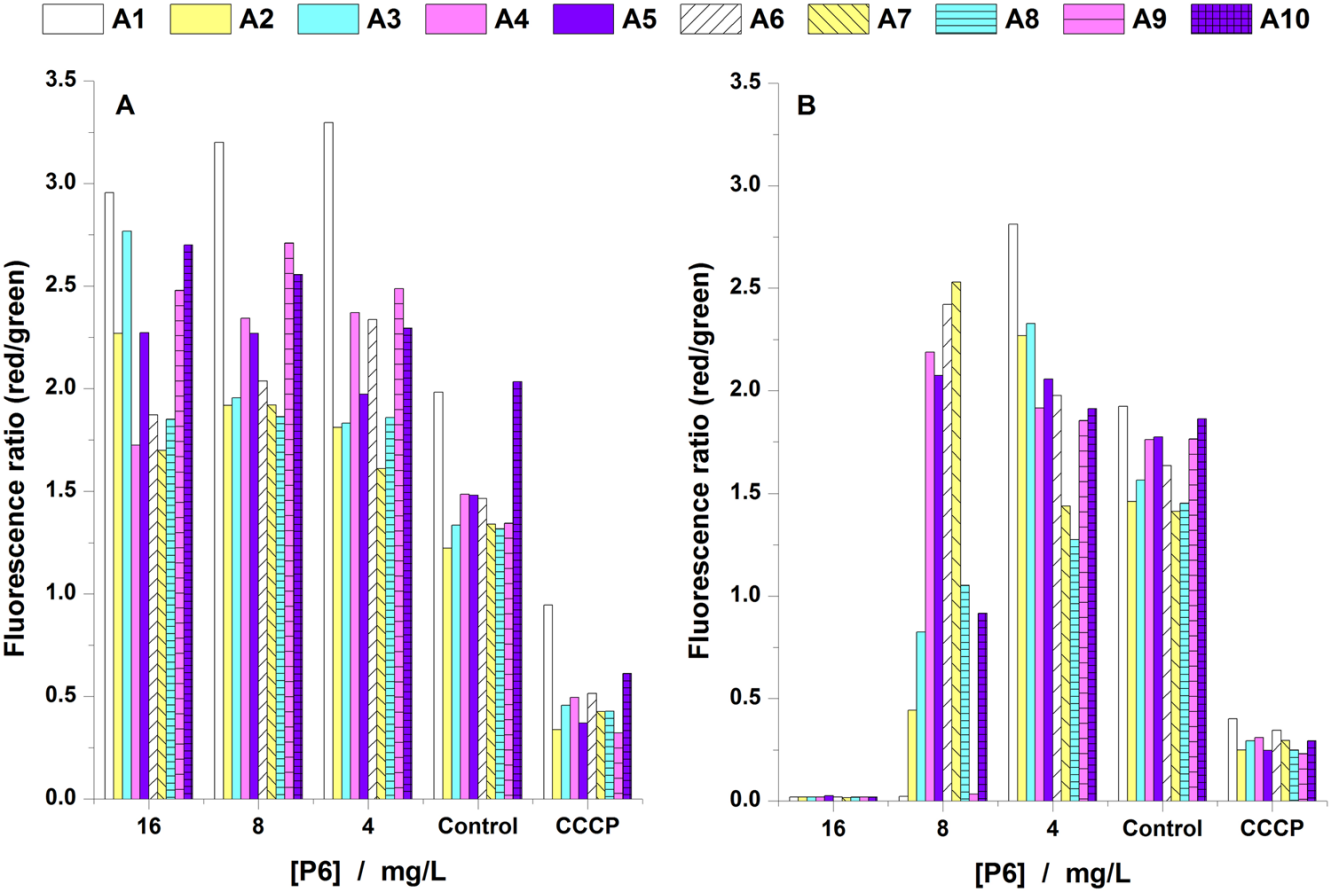
Ratios red/green fluorescence of DiOC_2_(3) probe for bacteria treated with P6 for 2 h (**A**) and 24 h (**B**).

Changes in membrane potential for all 10 clinical bacterial strains when treated for 2 h (Fig. 3A) or 24 h (Fig. 3B) with P6 at concentrations of 16, 8 and 4 mg/L (16 mg/L being the highest value of MIC, according data in Table 1) were monitored using the DiOC_2_(3) probe. The addition of P6 to the bacterial strains after 2 h did not induce any change to membrane potential for concentrations at MIC or below MIC. When the cells were treated for 24 h with the same concentrations of peptide, the ones treated at MIC are fully depolarized as compared with control and treated cells. The CCCP-ionophore treated cells (the positive control) show an intermediate value between the control cells and the cells treated with 16 mg/L P6. This indicates that at MIC the peptide adheres to the membrane and destabilizes the membrane potential by means of a slow mechanism.

### Localization of rhodamine-labeled peptides in bacteria

**Figure 4:**
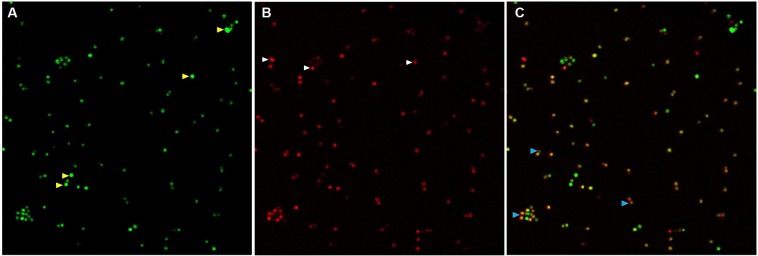
Localization of Rho-P6 in S. aureus: (**A**) S. aureus loaded with SYBR green, (**B**) S. aureus treated with 3x MIC Rho-P6 and (**C**) overlapping of the 2 images. Yellow arrows indicate the viable cells onto which the peptide did not attach, the white arrows indicate the dead cells with a high concentration of peptide attached and the blue arrows the cells that are still viable but to which the peptide started to attach to the membrane.

In order to determine the target site of P6, a reference strain of *S*. *aureus* was incubated with Rho-P6 at 3xMIC for 30 min and afterward with SYBR green marking the living cells. Fluorescence confocal imaging indicates the localization of Rho-P6 at the surface of the bacteria (Fig. 4). Live (green, Fig. 4A) and P6-attached cells (red, Fig. 4B) can be observed. Due to the short time of incubation, for some of the cells the green fluorescence co-localizes with the red one indicating the viable cells with surface attached peptides (blue arrows in Fig. 4C). Those cells only red in Fig. 4C are probably already dead. The fact that most of the cells are still alive after 30 min of incubation shows that the mechanism by which bacteria are killed is a slow one.

### Morphological changes by atomic force microscopy (AFM) technique

**Figure 5:**
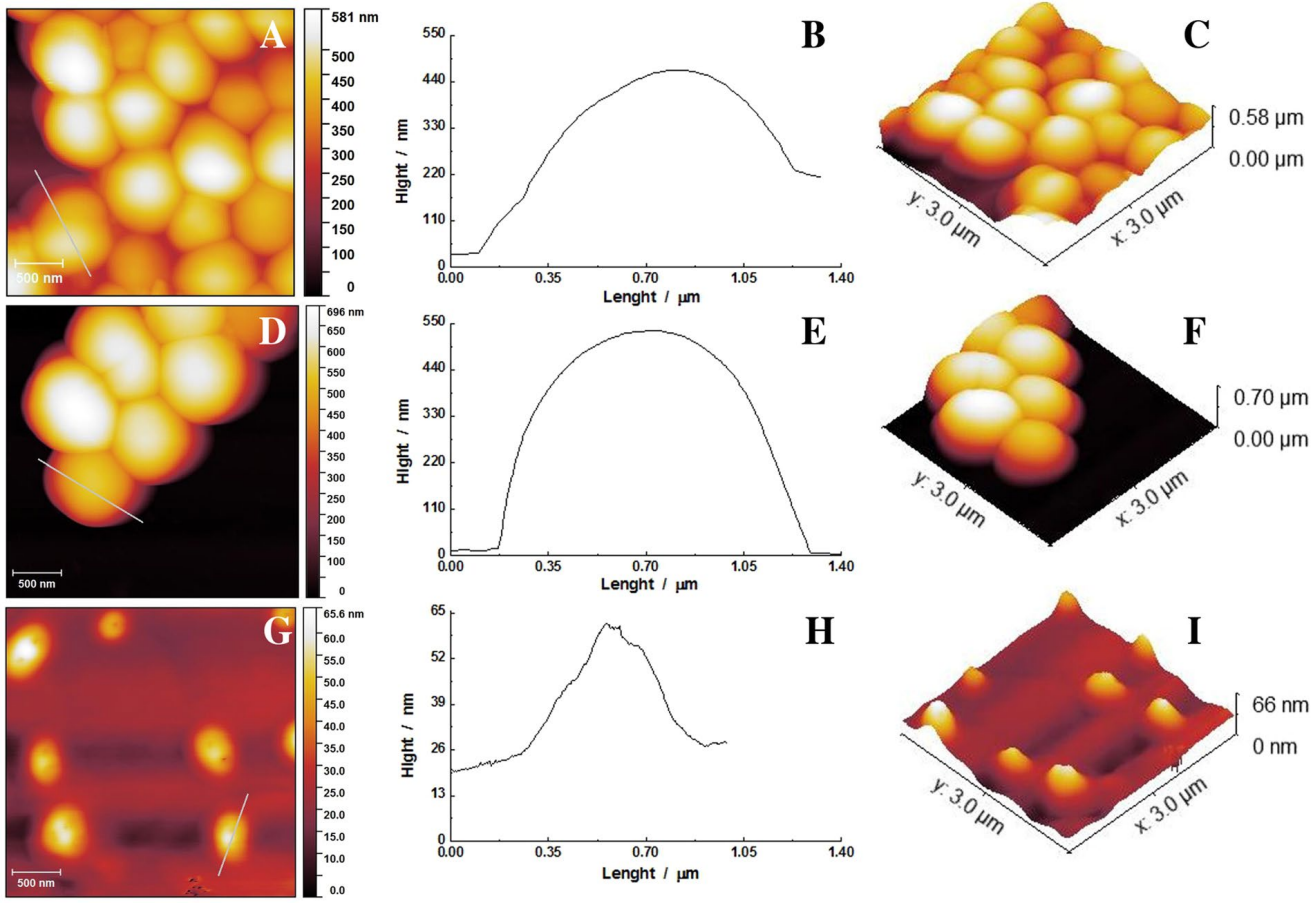
Morphological changes induced by P6 against A9 bacteria. Control (**A**), treated with MIC/2 (**D**) and with MIC (**G**) of P6: topography (**A**,**D**,**G**), cross section (**B**,**E**,**H**) and three dimensional (**C**,**F**,**I**) images.

AFM recordings were performed on 2 of the bacterial strains tested: A9, a MRSA strain having the most extended pattern of resistance to antibiotics and A6, a MRSA strain with no other resistance to additional classes of antibiotics. The bacterial cultures treated with MIC and MIC/2 concentrations of P6 and examined by AFM showed evidence of morphological changes induced at membrane level by peptide action (see Fig. 5 exemplified for A9).

The AFM images allowed us to determine and compare the bacterial size, but also provided details on bacterial cell surface topology. Untreated *S*. *aureus* shows a typical spherical morphology, with a relatively smooth surface and no alterations (Fig. 5A). The cell diameter is around 0.92 μm for A6 and 0.85 μm for A9, while the height for the two strains is around 400 nm (Fig. 5 and Table 2). The height was estimated through the cross-section profile for each bacteria recorded. The average roughness for the untreated cells is around 2.63 nm for A6 and 3.03 nm for A9.

**Table 2:** Morphological characteristics (diameter, height and roughness-R_a_) of A6 and A9 bacterial strains at MIC and MIC/2 of P6.

	A6			A9		
	Control	MIC/2	MIC	Control	MIC/2	MIC
Diameter/µm	0.92 ± 0.15	0.88 ± 0.14	1.04 ± 0.46	0.85 ± 0.14	0.89 ± 0.12	0.66 ± 0.16
Height/nm	425.95 ± 99.83	503.95 ± 74.28	301 ± 182.43	409.37 ± 71.81	416.96 ± 83.46	36.91 ± 14.25
R_a_/nm	2.63 ± 1.28	2.94 ± 1.53	3.87 ± 1.32	3.03 ± 1.67	3.59 ± 1.85	1.59 ± 0.69

When exposed to MIC/2 MRSA strains A6 and A9 are not affected by the treatment, all three parameters determined are not statistically different from the control (Fig. 5D–F). On the contrary, when treated with MIC, the bacteria are severely affected as observed in the surface topology images (Fig. 5). In Fig. 4G we can see that P6 treatment with MIC causes a decrease in height to ~37 nm and the diameter decreased to 0.66 µm. The image shows also structural changes caused by the formation of cavities and a decrease in average roughness to 1.59 nm. We cannot consider these changes as occurring to all the strains, since in the case of A6 only the height is significantly reduced while the diameter appears unchanged and the roughness is enhanced. Based on this we can expect that each strain has its own way to respond.

## Discussions

Over the last years, MRSA has become the most frequent cause of skin and soft tissue infections, dramatically increasing morbidity, mortality and finally the overall healthcare costs^22,23^. MRSA is currently one of the 12 categories of bacteria listed by WHO as a priority in researching and discovering new and better therapeutic methods^24^. A few alternative therapies were proposed against MRSA during recent years, including: natural antibacterial compounds, vaccines, prophylactic antibiotics, povidone–iodine, photodynamic therapy, etc^14,25^. One of the strategies addressed is the use of Antimicrobial Peptides (AMPs), particularly the cationic peptides. In a previous study we showed that P6, a synthetic cationic peptide rich in arginine and tryptophan residues, is active against both Gram negative and positive bacteria, including a reference *S*. *aureus* strain, in small concentrations, with low toxicity against human blood cells^20^. In order to better understand the action mechanism of P6 on *S*. *aureus* and to explore its potential in clinical application, in the present study we extended P6 examination against Community-Acquired MRSA.

The antimicrobial activity of P6 was evaluated against 10 clinical isolates from SSTIs in Romanian hospitals displaying a variety of resistance patterns^4^. The MRSA strains antibiotics resistance profile (including the virulence and resistance genes) have been characterized in a previous study^4^. Independent of the resistance pattern, the peptide was effective against all MRSA strains at similar concentrations as in the case of the control strain: MIC between 8–16 mg/L and MBC of 16 mg/L comparable with ~12 mg/L for the control strain^20^. Our results are in the same range (2–16 mg/L) as previous reports regarding the effects of short arginine and/or tryptophan rich peptides^16,18^, where the variation was observed between different *S*. *aureus* strains.

Considering that the bacterial strains were obtained from skin and soft tissue infections, we also tested the toxicity of P6 peptide against BJ human skin cells in order to determine its therapeutic potential for topical treatment. The toxicity on skin cells is reduced as compared with the toxicity we previously reported against human lymphocytes^20^, resulting a therapeutic index of ~30 that is in line with TI reported by previous studies regarding the effects of short arginine and/or tryptophan rich peptides^16,18^, TI varying between 16 and 128 depending on the bacterial strain. This result is encouraging for better exploring the possibility to use the P6 peptide as a therapeutic tool.

Considering the use of AMPs as therapeutics, a major limitation consists in their inactivation by binding to different biomolecules, such as proteins or lipids, or by interaction with serum proteases^26^. In order to assess such an effect, we evaluated the P6 efficiency against the reference strain in various conditions mimicking the *in vivo* conditions in systemic application. Thus, in the presence of 4% BSA our results (Fig. S1) revealed that P6 maintains both its bactericidal and bacteriostatic activity, but the concentration needed to obtain the same effect (32 mg/L) is 2 times higher than in the absence of BSA (16 mg/L). This result decreases the P6 TI at ~15 for systemic applications. To understand the causes of this effect we additionally studied the interaction of P6 with BSA alone, benefiting from the presence of tryptophan residues in both P6 and BSA structures. The fluorescence spectra of these molecules in solution look differently, the BSA fluorescence peak is blue shifted, Trp residue being embedded in the core of the protein tertiary structure. The P6 spectrum shift towards the BSA one in a few minutes when the molecules interact in the same solution proves the binding of P6 to some specific binding sites of BSA. This observation is supported also by the molecular simulation we performed showing that P6 binds to BSA binding sites with high affinity, all P6 being bound to BSA for the concentrations used in our experiments. These findings are in line with similar data reported in the literature regarding the binding of short synthetic AMPs on BSA (a calorimetry, NMR and molecular simulation study)^27^. These results suggest a limitation in the availability of P6 to interact with bacteria in the presence of BSA, such a mechanism explaining the increase in MIC and MBC values. Moreover, the drastic reduction in P6 antimicrobial efficacy in presence of salts or serum clearly indicate that P6 is not appropriate for systemic applications. Considering this result together with the reasonable TI value measured for skin fibroblasts we think that P6 can be a good candidate for topical application based therapies.

Indeed, understanding the mechanism through which P6 can kill bacteria is essential for developing it as a potential therapeutic. Because of its high number of tryptophan residues and positive charge, P6 is expected to interact strongly with bacterial membranes either by electrostatic or hydrophobic interactions as previously described in the literature for similar peptides^28–31^.

Because the bacteriostatic and bactericidal concentrations are almost similar for all strains tested, we evaluated the percentage of live and dead cells (using specific fluorescent markers) after treatment with P6 for 24 h for MIC (considered as being 16 mg/L) and lower concentrations. The P6 effects on strains prove a large variability of individual responses for lower concentrations, this variability vanishing at MIC, suggesting that there are details of P6 action mechanism differing from one strain to another. However, the incubation of the reference strain with a fluorescently labeled P6 (Rho-P6) indicates that the main target of the peptide is the bacterial membrane. After 30 min of incubation, most of the bacteria bound the Rho-P6 on the membrane but only few already died, suggesting a very slow mechanism of killing the bacteria. These suppositions are sustained by the AMF measurements. The large differences in responses recorded on two of the strains (A6 and A9) prove again the variability of the killing mechanism details. Even if 16 mg/L kills both strains, the size of the A6 cells (the strain with the largest resistance pattern) is less reduced compared with A9, the other two investigated parameters remaining almost unchanged.

Since P6 proved to bind to the surfaces of the bacteria we considered the possibility that membrane potential (MP) to be modulated by the P6 effects. Live bacteria, with an intact membrane and metabolically active, have a difference of electrical potential across the membrane, with the interior negative as compared with the exterior^32^. When applying different treatments the magnitude of the membrane potential can decrease (depolarizing the cell), can increase (hyperpolarizing the cell) or can be reduced to zero if it forms ruptures in the membrane (i.e. large holes through which inorganic ions can pass freely). When the membrane is ruptured, dyes like PI can also cross it. Although for some molecules the changes in the membrane potential and membrane permeability are correlated, there are also molecules (ionophores) known to affect only the membrane potential without altering the permeability to PI^32^.

In our study we tested the ability of P6 to affect the MP of MRSA strains using the fluorescent dye DiOC_2_(3)^33^. Our results show that P6 effect on MP is almost negligible after 2 h of treatment, but after 24 h it induced a dose-dependent membrane depolarization for the tested bacterial strains (Fig. 3). However, the fluorescently labeled P6 (Rho-P6) showed binding of the P6 on the membrane surface in less than 30 min. This suggests that P6 dissipated MP through a slow mechanism. The data can be discussed also in correlation with bacterial growth curves (Fig. S1A) and live/dead assay (Fig. 2A). For all concentrations tested (0 to 64 mg/L), in the first 2–3 h after the peptide was added, one can observe a small growth of bacteria. These results correlated with the MP experiments prove the slow action mechanism of P6. Only after 5 h bacterial growth was inhibited for concentrations higher than 8 mg/L. After 24 h only the concentrations between 16 and 64 mg/L inhibited the growth of bacteria over the entire treatment period. If we check the percentage of live cells for bacteria treated for 24 h with 16 mg/L and 8 mg/L we have around 5% and 30% respectively, as compared to control (Fig. 2A). Consequently, MP almost vanish at 16 mg/L while at 8 mg/L we have bacterial strains with unaffected MP but also strains for which MP decreased to half as compared to control. This suggests that the bactericidal effect of P6 is correlated with a gradual dissipation of the membrane potential in MRSA, the delay in affecting the MP differing from one strain to another. To explore more this issue, a time dependence of MP was recorded over the first 4 h after adding the peptide (results in Supplementary materials, Fig. S2) the findings being consistent with previous observations of slow mechanism of MP dissipation by P6.

Based on our findings, we conclude that the action mechanism of P6 is different as compared with other antimicrobial peptides that can depolarize faster the membrane^31,34,35^. Considering also our molecular simulation from previous work that did not prove any tenancy of P6 to penetrate a lipid bilayer (only an adherence process was observed^20^), we may speculate that P6 do not form pores into bacteria membrane, appearing to follow “the Interfacial Activity Model” introduced by Wimley in his review regarding the AMP action mechanisms^36^ rather than a standard pore model. Even the most studied short Trp-rich peptide, indolicidin, was proved to have a low capacity to permeabilize the bacteria membrane or to depolarize it^37^. Therefore, a complex action mechanism with a slow evolution may be present. Such a mechanism may include an interaction of the peptide with the membrane external surface molecular constituents interfering with the membrane bacteria activity as described for other short peptide as organometallic AMP^38^ or short Arg and Trp rich peptides (RWRWRW-NH_2_). The last have been proved to produce delocalization of peripheral membrane proteins with major consequences on bacteria metabolism^39^.

## Conclusion

P6 proved to be efficient against clinical *S*. *aureus* strains, including Methicillin-Resistant *Staphylococcus aureus* isolated from skin and soft tissue infections, at low concentrations and with reduced toxicity against eukaryotic skin cells. It is highly probable that P6 can kill bacteria by attaching to the membrane, the depolarization and death of bacteria being a slow process. Due to its promising TI, but considering the inhibition produced by salts and serum, P6 may become a good candidate for use as an antimicrobial agent in topical applications and extended to *in vivo* studies laboratories and clinical trials.

## Material and Methods

### Bacterial strains, reagents and media

A total of 10 clinical *Staphylococcus aureus* strains (9 MRSA and one MSSA) and *S*. *aureus* ATCC 25923 reference strains stored at −80 °C in Brain Heart Infusion (BHI) Broth with 50% glycerol were cultivated on Columbia Blood Agar Base (Oxoid) supplemented with 7% defibrinated sheep blood and incubated at 35 ± 2 °C for 18–24 h. Clinical *S*. *aureus* strains have been isolated in a previous study from patients with SSTIs and referred for laboratory investigations and research to “Cantacuzino” National Institute Reference Laboratory^4^. All subjects provided written informed consent. Clinical strains have been isolated using a protocol approved by the Local Ethics Committee of Cantacuzino Institute (no. CE/31/06.05.2014). All methods used in this study were performed in accordance with the relevant guidelines and regulations in this research field.

The taxonomic affiliation of all 10 isolates has been confirmed by a combination of phenotypic and genotypic tests: bound and free coagulase and PCR for the *nuc* gene detection^4^.

Peptide P6 synthesis and characterization were reported in our previous study, and we prepared the peptide solution from the initial batch synthetized^20^. All cell cultivation media and reagents were purchased from Biochrom AG (Berlin, Germany). Mueller-Hinton broth, Mueller-Hinton agar and saline solution were purchased from Merck KGaA (Darmstadt, Germany).

### Determination of minimum inhibitory concentration (MIC)

The P6 antimicrobial activity was assayed according to ISO 20776–1 (2006) recommended for the detection of MIC by the European Committee for Antimicrobial Susceptibility Testing (EUCAST). In brief, two-fold serial dilutions of P6 peptide in Mueller Hinton Broth were placed in a microwell titer plate and peptide charged wells were then inoculated with bacteria, at a final concentration of 10^5^ CFU/mL. MICs were tested against the reference strain (*S*. *aureus* ATCC 25923) and the 10 clinical *S*. *aureus* SSTIs strains. The final peptide concentrations were in the range of 32 to 4 mg/L. Two types of controls were used to check our procedure, namely Growth Control (GC) and Sterility Control (SC). The plate was covered with a protective foil and incubated at 35 ± 2 °C for 18–24 h.

### Determination of minimum bactericidal concentration (MBC)

The minimum Bactericidal Concentrations of P6 against the same bacterial strains were determined by sub-culturing 10 µL of broth from the endpoint well and the next two higher dilutions of the peptide, on Columbia Blood Agar Base (Oxoid) supplemented with 7% defibrinated sheep blood. The MBC was regarded as the minimal concentration that prevented any residual colony from forming.

### Cytotoxicity assay

Cytotoxicity was assessed by using the tetrazolium salt MTT dye (Serva) following the instruction from the manufacturer. Briefly, BJ cells (30,000 cells/well) were seeded in 96 well plates and cultured for 24 h in Minimum Essential Medium (MEM) supplemented with 2 mM L-Glutamine and 10% fetal calf serum (FCS). After this, the cells were treated with different concentrations of P6. Untreated cells were used as negative control. After 24 h, the cells were washed, the medium was changed and 20 µL of MTT dye were added in each well and incubated at 37 °C for an additional interval of 4 h. The medium was thereafter collected and the crystals were dissolved in DMSO. The absorbance of the samples was recorded at 570 nm using a Mithras 940 (Berthold, Germany) plate reader. The data were corrected against the background and then the percentage of cell viability was calculated as follows:

$$ \% \,{\rm{cell}}\,{\rm{viability}}=({\rm{absorbance}}\,{\rm{of}}\,{\rm{treated}}\,{\rm{cells}}/{\rm{absorbance}}\,{\rm{of}}\,{\rm{untreated}}\,{\rm{cells}})\times 100.$$

### Live/dead assay

LIVE/DEAD BacLight kit (Invitrogen) was used according to the protocol supplied by the manufacturer. Propidium Iodide (PI) and cyanine based organic SYTO9 dye were loaded for 15 min into bacteria, in the dark, after all clinical strains were treated for 24 h with different concentrations of P6. The samples fluorescence was recorded for PI (excitation: 540 nm/emission: 630 nm) and SYTO9 (excitation: 485 nm/emission: 530 nm) using a Tecan Infinite M1000 microplate reader (Tecan Group Ltd., Mannedorf, Switzerland).

### Membrane depolarization assay

Membrane potential was determined using the carbocyanine dye DiOC_2_(3) (Invitrogen) as described hereinafter. The bacteria were first incubated at 37 °C with variable concentrations (4, 8 and 16 mg/L) of the tested peptide. After 2 h or 24 h of incubation with peptides, we added the protonophore CCCP (Invitrogen), at a final concentration of 5 μM, in a well with non-treated cells and maintained it in contact with the cells for 15 min, to be used as a depolarized positive control. Finally, 30 μM DiOC_2_(3) were added in all wells and let to incubate for 30 min. All samples were recorded using a Tecan Infinite M1000 microplate reader (excitation at 485/10 nm and fluorescence at 530/10 nm and 630/10 nm) and membrane potential was quantified by means of the red over green fluorescence ratio. When found in the presence of intact bacterial membranes, the fluorescence of the dye shifts from green to red, while in the case of the depolarized membranes the shifts disappears^32^. Although monitoring only the red fluorescence is sufficient to assess the changes in the MP, calculating the red to green ratio allows for a more accurate estimation of the MP changes.

### Localization of rhodamine-labeled peptides in the bacterial cell

To check the cellular localization of P6, the reference strain was incubated with 3x MIC of Rhodamine-P6. After incubation for 30 min, the cells were washed with PBS buffer, pre-incubated with SYBR Green and then observed by confocal microscopy using a DSD2 device (Andor, UK) mounted on an upright microscope, BX51 (Olympus, Germany). Recordings were performed using the FITC and TRICT filters.

### Morphological changes by atomic force microscopy (AFM) technique

The morphological changes of two of the tested strains (A6 and A9) were analyzed by AFM using a NanoWizard 4 BioScience AFM (JPK instruments AG, Germany). The protocol was used as previously described^20^. Briefly, bacterial strains were treated as previously described, in the presence of the peptide at MIC and MIC/2, for 18–24 h, with continuous agitation. Then, the cells were centrifuged, washed in sterile phosphate buffer saline and re-suspended in deionized sterile water. Finally, cell suspensions (10 μL of each condition) were applied, on circular mica disks (V1 Ruby Muscovite) and left to dry prior to AFM imaging. An area of 4 μm × 4 μm was scanned and the images were analyzed with Gwyddion software to obtain the morphological characteristics (diameter, height and roughness-Ra).

## Supplementary information

Supplementary material
