# Supplementary material for: Short tryptophan- and arginine-rich peptide shows efficacy against clinical methicillin-resistant Staphylococcus aureus strains isolated from skin and soft tissue infections
Source: Sci Rep. 2019 Nov 20;9:17176. doi: 10.1038/s41598-019-53926-4 (PMC6868180; doi:10.1038/s41598-019-53926-4)
Supplement: Supplementary file 1 — Supplementary material [file 41598_2019_53926_MOESM1_ESM.docx]

Supplementary data

Short tryptophan- and arginine-rich peptide shows efficacy against clinical methicillin-resistant *Staphylococcus aureus* strains isolated from skin and soft tissue infections

Mihaela Bacalum^1^, Elena-Carmina Dragulescu^2^, George Necula^3^, Irina Codita^2, 4^, Mihai Radu^1^

*^1^Department of Life and Environmental Physics, “Horia Hulubei” National Institute of Physics and Nuclear Engineering, Măgurele, Romania,*

*^2^Nosocomial and Antimicrobial Resistant Infections Laboratory, “Cantacuzino” National Medico-Military Institute for Research and Development, Bucharest, Romania*

*^3^Department of Computational Physics and Information Technologies, “Horia Hulubei” National Institute of Physics and Nuclear Engineering, Măgurele, Romania,*

*^4^“Carol Davila” University of Medicine and Pharmacy, Bucharest, Romania*

Corresponding author: Mihai Radu

E-mail: mradu@nipne.ro

**Material and methods**

1. **Bovine Serum Albumin (BSA), Fetal Bovin Serum (FBS) and salts inhibition effect on peptide activity**

To study the effect of BSA (Sigma-Aldrich, Germany) on the killing kinetics of the peptide, a time-kill assay was performed for concentrations of peptide between 64 to 4 mg/L alone or in the presence of 4% BSA. The absorbance at 600 nm was recorded over a period of ~24 h using a SpectraMax i3x Multi-Mode Microplate Reader (Molecular Devices, USA). Also, two different concentrations of FBS (25% and 50%) and NaCl (100 mM and 200 mM) were used to investigate MIC values for the standard strain.

1. **Fluorescence spectroscopy of P6 and BSA interaction**

Steady-state fluorescence measurements of P6 and BSA were performed using a FluoroMax 3 spectrofluorimeter (Horiba Jobin Yvon, Edison, NJ, USA) equipped with a Peltier thermostated cell holder. The excitation wavelength was set at 284 nm, and the spectra were recorded between 300 to 400 nm, with excitation and emission slits set at 3 nm. The spectra recorded were first corrected for the spectral sensitivity of the emission channel of the spectrofluorimeter and finally for Raman and scattering artifacts by subtracting from the spectra the contribution of the buffer.

1. **Computational Docking**

The crystal structure of BSA was retrieved from the Research Collaboratory for Structural Bioinformatics Protein Data Bank (RCSB PDB) (code 3V03). The structure of BSA was prepared for computational docking with CHARMM v41 program [^1^](#_gjdgxs). The structure of antimicrobial peptide P6 was built with Molefacture plugin v1.3 of VMD version 1.9.3 program [^2^](#_30j0zll). The BSA structure was formatted to AutoDock4 atom types (adding Gasteiger charges, merging non-polar hydrogens and detecting aromatic carbons), and was also applied for P6 ligand structure with the addition of specifying 44 rotatable bonds. Receptor and ligand structures were formatted with PyRx version 0.8 program that submitted the AutoDock Vina [^3^](#_1fob9te) computational docking with default parameters. The representation of the docking complex was prepared in PyMOL version 1.8.2 [^4^](#_3znysh7).

1. **Membrane depolarization kinetics**

To investigate the kinetic of the membrane we used 2 μM Bis-(1,3-dibutylbarbituric acid) trimethine oxonol [DiBAC_4_(3)] (Invitrogen) that is recommended for longer recordings since do not inhibit the bacteria growth [5]. Briefly, 2 μM DiBAC4(3) were added in all wells and allowed to incubate for 10 min. The signal was recorded (Tecan Infinite M1000 microplate reader: excitation at 485/10 nm and fluorescence at 530/10 nm) and then the bacteria were incubated with variable concentrations (4, 8 and 16 mg/L) of the tested peptide and recorded for 4h each 5 min.

**Results**

**BSA inhibition effect on peptide activity**

Serum inhibition on the antimicrobial activity of the peptide was tested in the absence/presence of 4% BSA on the reference strain (*S. aureus* ATCC 25923). In figure S1 we present the bacterial growth curves for different peptide concentrations in the presence of BSA (Fig. S1B) compared with the control experiment (absence of BSA - Fig. S1A). In the absence of BSA, peptide concentrations of 64, 32 and 16 mg/L P6 inhibited the growth of bacteria throughout the experimental observation. For the concentration of 8 mg/L, bacterial growth was inhibited for ~12 h after which the bacteria entered the growing phase. The concentration of 4 mg/L did not affect the growing of bacteria. In the case of 4% BSA, only the concentration of 64 and 32 mg/L of P6 inhibited the growth of bacteria throughout the experimental observation, while concentrations of 16 mg/L or lower did not affect the growing of bacteria.

In order to check the interaction between 1.5 mg/L P6 and 13 mg/L BSA we recorded the fluorescence emission spectra of the two molecules alone or together (Fig. S1C). When the two molecules are alone in PBS the emission maxima are around 349 nm for P6 and 338 nm for BSA. The differences in the spectra are determined by the environment in which the tryptophan residues are found. Thus, for P6 all 4 tryptophan residues are exposed to water and fluorescence emission is found at longer wavelengths as compared with the BSA tryptophan’s which are less exposed to water, thus emitting at a smaller wavelength. When the two molecules are placed together we can observe almost a blue shift in the emission maximum of the spectra recorded (341 nm). This is due to the presence of BSA with which the P6 molecules interact, presumably with one of the binding sites, thus decreasing the exposure of tryptophan residues to water. After 15 min the emission maximum of the complex is 338 nm, similar to the one obtained for BSA alone, suggesting that all the P6 molecules are bound to BSA.


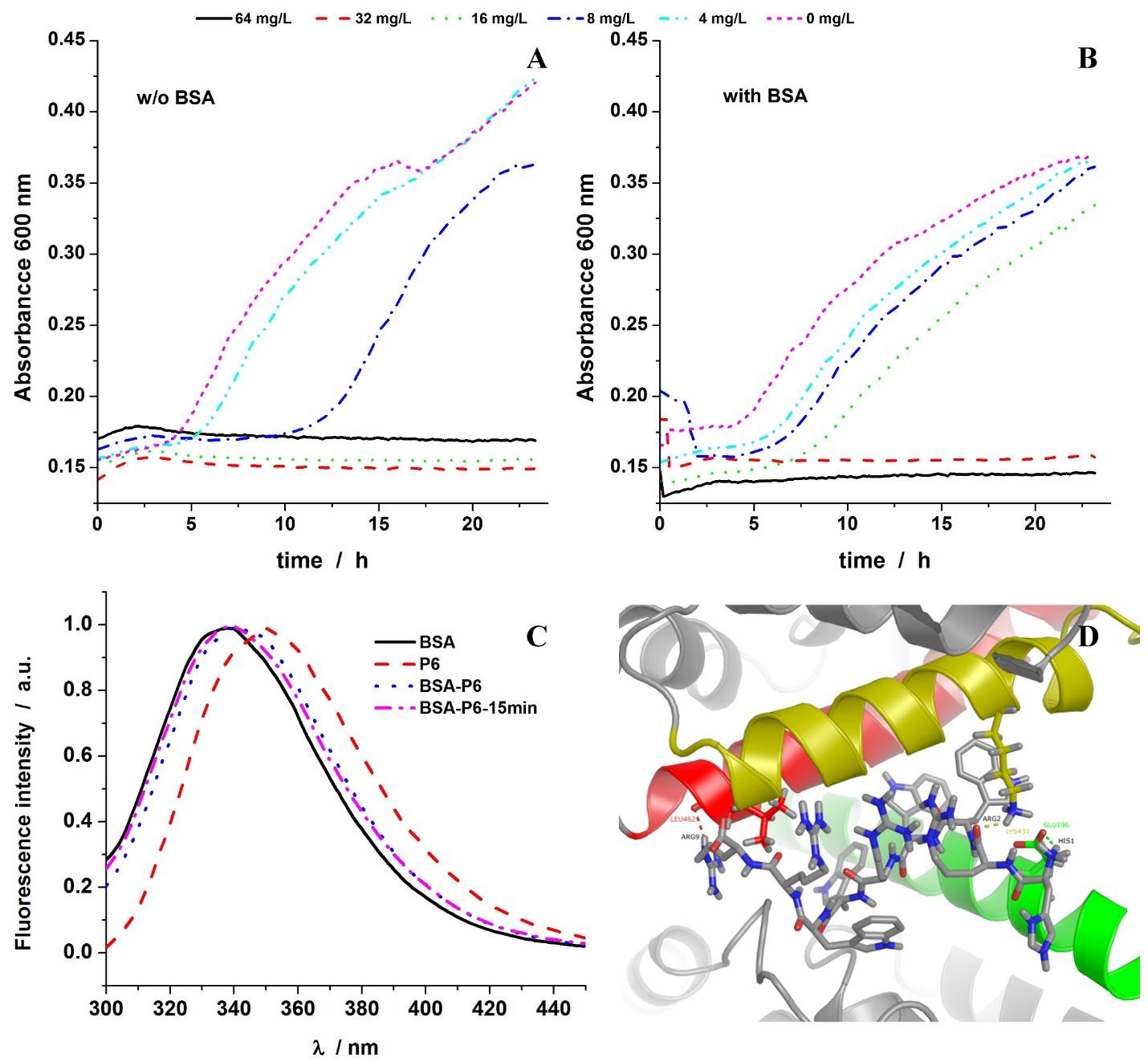


*Figure S1. Growing curves of bacteria in the presence of different concentrations of P6 in the absence (A) and presence (B) of 4% BSA. (C) Fluorescence spectra of BSA and P6 alone or together (D) Docked pose of P6 (ball and sticks) inside BSA structure (ribbons). Three subdomains of BSA are highlighted: IIIA-h3 (yellow color), IIIA-h4 (red color), and IB-h4 (green color). Hydrogen bonds are represented with dotted lines. This figure appears in colour in the online version of JAC and in black and white in the print version of JAC*

Using the molecular docking analysis we checked the interactions mechanism between the two molecules. Figure S1D shows the P6 peptide slots in a space between domains IIIA and IB of BSA, more precisely intercalated between subdomains IIIA-h3 (yellow alpha-helix), IIIA-h4 (red alpha-helix) and IB-h4 (green alpha-helix). The top three docking poses bind very similarly to BSA, and with lower than or equal to -10 kcal/mol binding energy (-10.5, -10.1, -10 kcal/mol). Three hydrogen bonds are formed between the docking pose with the lowest binding energy (-10.5 kcal/mol) and binding site residues of BSA: the first formed between ARG9 of P6 and LEU462 (2.1 Å) from subdomain IIIA-h4, the second between HSD1 and GLU186 (2.1 Å) located in subdomain IB-h4, and the third formed by LYS431 (subdomain IIIA-h3) and ARG2 (2.0 Å) Fig. S1D. The docking pose with the lowest binding energy also establishes two pi-cation interactions with BSA binding site: the first with the side chain nitrogen of LYS431 and indole moiety of TRP3, and the second between the nitrogen of LYS114 (located on the loop connecting subdomains IA-h6 and IB-h1) and imidazole side chain of HIS1. The indole moiety of P6 residues TRP3 and TRP4 are buried in small hydrophobic pockets: TRP3 makes hydrophobic contacts with VAL432, LEU189 and ILE455, while TRP4 is in contact with hydrophobic residues ALA193, LEU189, LEU454, ILE455 and TYR451. Residue TRP6 is in close contact with hydrophobic residue PRO110 and also with positive charged residues ARG458, ARG196, ARG144 and HSD145.

**Membrane depolarization kinetics**

For simplification, in Figure S2 are represented the kinetic curves only for the highest concentration tested (16 mg/L) and one control. The recordings show that the membrane potential is lost slowly. Some of the bacteria show a faster phase of ~ 0.5 h, but for others this phase appears to be longer, ~2 h, and the signal is still slowly increasing after this phase. For comparison, in other antimicrobial peptides studies the potential is lost in tens of seconds [6]. For the bacteria treated with 8 and 4 mg/L P6 the membrane potential was less affected, or not affected at all by the treatment (data not shown).


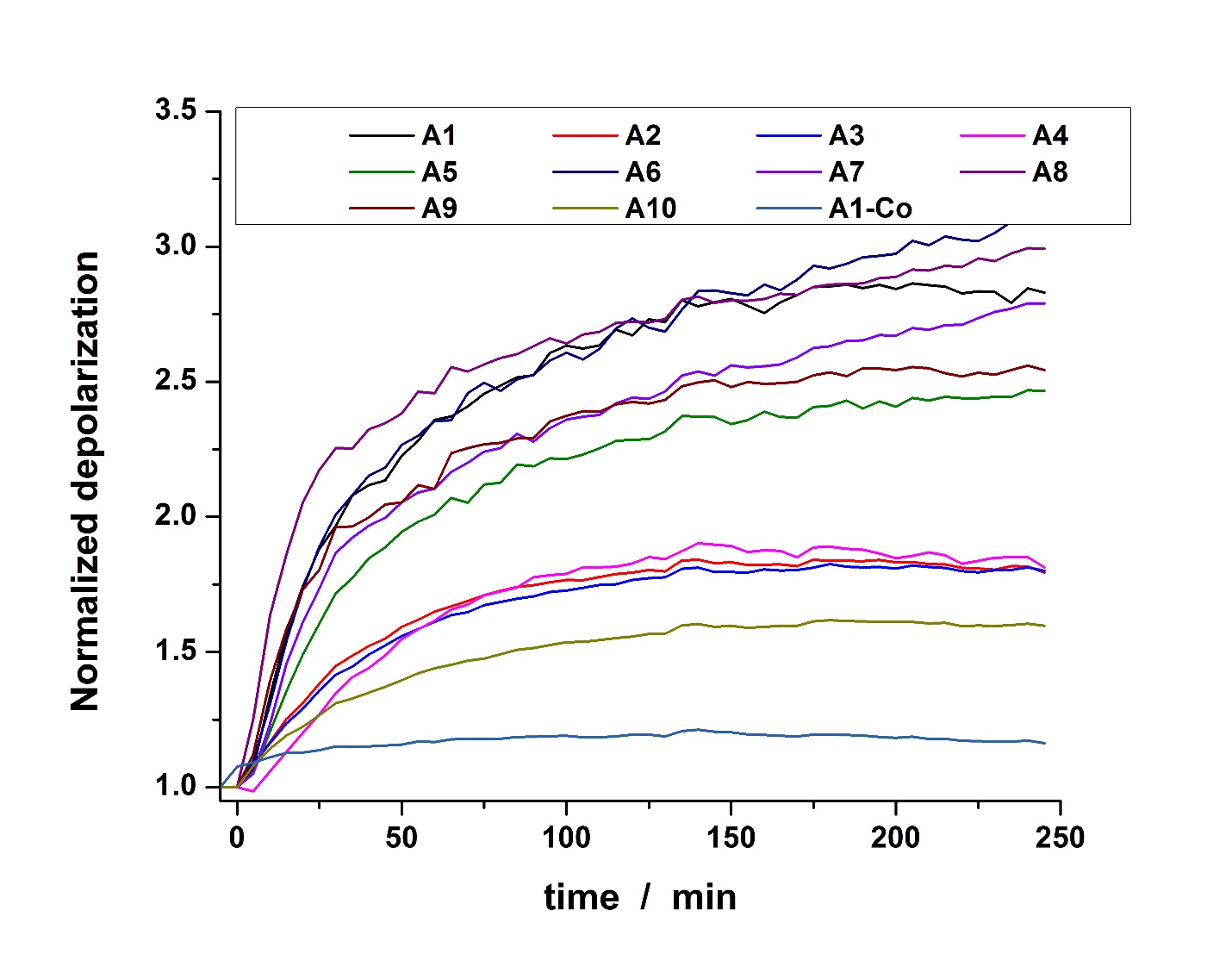


*Figure S2. Bacterial membrane potential (fluorescence intensity normalized at the value stabilized after 10 min; the 0 moment in the graph is that of adding the P6 peptide) of the 10 resistant strains monitored by the DiBAC4(3) for 4h after 16 mg/L P6 was added. For simplification, only one of the controls is represented in the graphic.*

**References**

1 Brooks, B. R. *et al.* CHARMM: The Biomolecular Simulation Program. *J Comput Chem* **30**, 1545-1614, doi:10.1002/jcc.21287 (2009).

2 Humphrey, W., Dalke, A. & Schulten, K. VMD: visual molecular dynamics. *Journal of molecular graphics* **14**, 33-38, 27-38 (1996).

3 Trott, O. & Olson, A. J. Software News and Update AutoDock Vina: Improving the Speed and Accuracy of Docking with a New Scoring Function, Efficient Optimization, and Multithreading. *J Comput Chem* **31**, 455-461, doi:10.1002/jcc.21334 (2010).

4 DeLano, W. L. The PyMol Molecular Graphics System. *Proteins Structure Function and Bioinformatics* **30**, 442-454 (2002).

5 Derk te Winkel J. *el at*. Analysis of Antimicrobial-Triggered Membrane Depolarization Using Voltage Sensitive Dyes. *Frontiers in Cell and Developmental Biology* **4**, 29 (2014)

6 Yasir M *et al.*, Comparative mode of action of the antimicrobial peptide melimine and its derivative Mel4 against Pseudomonas aeruginosa. Scientific Reports **9**, 7063 (2019)
